# Prognostic value of the albumin-bilirubin score in patients with non-Hodgkin lymphoma-associated hemophagocytic lymphohistiocytosis

**DOI:** 10.3389/fimmu.2023.1162320

**Published:** 2023-05-17

**Authors:** Wanying Cheng, Limin Duan, Ji Xu, Yongqian Shu, Hongxia Qiu, Guangli Yin

**Affiliations:** ^1^ Department of Geriatric Hematology, The First Affiliated Hospital of Nanjing Medical University, Jiangsu Province Hospital, Nanjing, China; ^2^ Department of Hematology, Wuxi People’s Hospital, Nanjing Medical University, Wuxi, China; ^3^ Department of Oncology, The First Affiliated Hospital of Nanjing Medical University, Jiangsu Province Hospital, Nanjing, China

**Keywords:** hemophagocytic lymphohistiocytosis, non-Hodgkin lymphoma, ALBI score, ferritin, hepatic injuries

## Abstract

Secondary hemophagocytic lymphohistiocytosis (sHLH) is a rare life-threatening systemic disease. This study aimed to assess the prognostic value of pretreatment albumin-bilirubin (ALBI). We retrospectively analyzed 168 non-Hodgkin lymphoma-associated secondary hemophagocytic lymphohistiocytosis (NHL-sHLH) patients with hepatic injuries. Multivariable logistic/Cox models and restricted cubic spline models were conducted to evaluate the relationships between the ALBI score and short- and long-term survival. Among 168 adult NHL-sHLH patients, 82 (48.8%) patients died within 30 days after admission, and 144 (85.7%) patients died during the follow-up period. Multivariable logistic/Cox regression model indicated that ALBI grade could be an independent risk factor for predicting the prognosis of patients with 30-day mortality and overall survival (odds ratios [OR]_30 days_ 5.37, 95% confidence interval 2.41-12.64, *P* < 0.001; hazard ratios [HR]_OS_ 1.52, 95% confidence interval 1.06-2.18, *P* = 0.023), respectively. The restricted cubic spline curve displayed a linear and positive relationship between the ALBI score and risk of mortality (*P* for nonlinearity =0.503). Furthermore, receiver operating characteristic (ROC) curve analysis showed that the area under the curve (AUC) for predicting mortality by integrative analysis of the ALBI score and ferritin was significantly improved compared to the ALBI score (AUC _30 days_: 0.820 vs 0.693, *P* = 0.001; AUC_1 year_: 0.754 vs 0.681, *P* = 0.043) or ferritin (AUC_30 days_: 0.820 vs 0.724, *P* = 0.005; AUC_1 year_: 0.754 vs 0.658, *P* = 0.031) alone. The ALBI score could be a useful indicator of short and long-term survival for NHL-sHLH patients with hepatic injuries.

## Introduction

Secondary hemophagocytic lymphohistiocytosis (sHLH) is a rare life-threatening systemic disease characterized by the uncontrolled activation of T cells and macrophages, producing overwhelming hypercytokinemia and leading to multiple organ failure (1). sHLH is often associated with neoplastic diseases, including lymphoma, viral infections, and autoimmune or autoinflammatory disorders (2). Of these, sHLH in the context of lymphoma is considered a major challenge to clinicians due to variable overlaps of symptoms, such as hepatic dysfunction and multiorgan failure, thus resulting in a higher incidence of mortality (3). Although serum ferritin has been validated to predict outcomes (4, 5), it is not refined enough to evaluate prognosis in non-Hodgkin lymphoma-associated sHLH (NHL-sHLH) patients with hepatic injuries. Hepatic injuries, which present with a wide range of hepatic dysfunction, are recognized as one of the major complications in patients with sHLH and sometimes cause acute liver failure (ALF), which can contribute to high syndromic mortality (6, 7). In a previous report of 12 HLH patient autopsies, 75% (9/12) of patients had evidence of hepatocellular injury, and all autopsies revealed significant liver injury, including necrosis, fibrosis, and cholestasis (8).

However, sHLH at presentation is usually accompanied by coagulopathy, and liver biopsy cannot be routinely obtained to assess liver injury (9). It is therefore necessary to develop a tool to assess the severity of hepatic dysfunction. Recently, the albumin-bilirubin (ALBI) score/grade was proposed to predict liver dysfunction and survival status among patients with hepatocellular carcinoma but was also validated in patients with liver cirrhosis without nonhepatocellular carcinoma, acute heart failure, and acute pancreatitis (10–13). Moreover, the levels of serum albumin and total bilirubin can previously reflect liver function and prognosis in sHLH patients (14).

Using this background, we conducted the present study to evaluate the ALBI score/grade and potential predictive risk of patients with NHL-sHLH with hepatic injuries.

## Methods

### Study patients

The clinical and laboratory data were extracted from the hospital database of 168 consecutive NHL-sHLH patients with hepatic injuries at our center from February 1, 2014, to February 1, 2019, for this retrospective study. All the enrolled patients for whom the new pathological diagnosis of NHL met WHO pathological criteria for biopsy samples (15) or patients for whom the diagnosis of NHL was based on MICM (morphology, flow cytometric immunophenotype, IgH or TCR rearrangement and immunohistochemistry) of bone marrow biopsy criteria (16). Meanwhile, NHL patients were assessed to confirm HLH diagnosis by the HLH-2004 diagnostic criteria of the Histiocyte Society (17) and HScore (18) before treatment. The exclusion criteria were as follows: 1) patients who were diagnosed with nonlymphoma-associated sHLH (n=87) and Hodgkin lymphoma-associated sHLH (n=4); 2) patients who had no hepatic injuries (normal serum aminotransaminase, albumin, bilirubin levels, and no hepatomegaly) (n=22); 3) patients who had a history of hepatitis B, hepatitis C, cirrhosis or other serious liver diseases with decompensated liver function as well as ascites and jaundice; 4) patients who had missing data on albumin and bilirubin variables (n=5); and 5) patients who had no follow-up data (n=3). Our study was approved by the Ethics Committee of the First Affiliated Hospital of Nanjing Medical University (Clinical Trial: ChiCTR2000032421), in accordance with the guidelines of the 1975 Declaration of Helsinki. Informed consent was obtained to review patient medical records.

### Calculation of ALBI score and definition of hepatic injury

The ALBI score was calculated before treatment using appropriate clinical parameters and recommended methods (19). The ALBI score was calculated by the formula: (0.66 × log_10_ bilirubin) – (0.085 × albumin). ALBI grades were defined as ALBI grade 1 (score ≤ -2.60), ALBI grade 2 (score > -2.60 and ≤ -1.39) and ALBI grade 3 (score > -1.39). During calculation, serum bilirubin was expressed in μmol/L, and serum albumin level was expressed in g/L (**Supplementary Figure 1**). According to previous literature reports, hepatic injury/dysfunction was defined as at least two of the following: 1) elevated bilirubin, 2) elevated alanine and aspartate aminotransferase (ALT, AST) levels, 3) low albumin levels, 4) coagulopathy and 5) hepatomegaly (9, 20).

### Covariate collection and follow-up

Patients’ clinical characteristics assessed at the first admission included age; sex; fever; complete blood cell counts; hepatosplenomegaly; and blood biochemical tests (including albumin, bilirubin, aspartate aminotransferase (AST), alanine aminotransferase (ALT), triglycerides (TG), lactate dehydrogenase (LDH)), fibrinogen (FIB), ferritin, and serum soluble interleukin-2 receptor (sIL-2R, sCD25) were reviewed from their medical records on admission. Epstein-Barr virus (EBV), viruses C and B were evaluated by both serology and EBV DNA real-time quantitative polymerase chain reaction (RQ-PCR) analysis. The NK-cell cytotoxicity assay is not available at our facility. Bone marrow aspiration and biopsy samples were reviewed at the first diagnosis. The primary outcome of the current study was overall survival (OS), which was calculated as the time in days from NHL-sHLH first diagnosis to the date of death from any cause or the last follow-up to June 2020. The secondary outcome was 30-day mortality, which described the survival status in the first 30 days on admission.

### Treatments

After the diagnosis was confirmed, patients were reviewed at our multidisciplinary HLH team for treatment recommendations. Among these 168 patients, 124 patients (71.9%) had received various kinds of chemotherapy, including 62 patients treated with a CEOP ± R-based regimen (cyclophosphamide, vincristine, etoposide, prednisone, and/or rituximab), 23 treated with the SMILE regimen (dexamethasone, methotrexate, ifosfamide, L-asparaginase, and etoposide), 18 treated with the DEP regimen (liposomal doxorubicin, etoposide, and methylprednisolone), 14 treated with the LMED regimen (methotrexate, etoposide, L-asparaginase, and dexamethasone), 4 treated with the P-GemOx regimen (pegaspargase, gemcitabine, oxaliplatin), 2 treated with anti-programmed death 1 antibody, and 1 treated with a modified hypercytbine-CVAD regimen (cyclophosphamide, doxorubicin, vincristine and dexamethasone alternating with rituximab, high-dose methotrexate and cytarabine). A median of 1 cycle (range 1–6 cycles) was given. The other 44 patients received only the HLH-94 protocol or steroid and/or etoposide and intravenous immunoglobulin therapies.

### Statistical analysis

Continuous variables are presented as the mean ± standard derivation or median (with interquartile range). Category variables are expressed as frequencies or percentages. The comparison of survival probability was performed by the Kaplan‐Meier method with the log‐rank test. Univariable logistic regression and Cox proportional hazards models were used to estimate odds ratios (ORs)/hazard ratios (HRs) and 95% confidence intervals (95% CIs) for associations between clinical and laboratory prognostic factors and 30-day mortality as well as overall survival outcomes. Multivariable regressions with stepwise forward selection were performed to analyze the influence of relevant variables (variables with *P <*0.05 in univariable Cox regression were subsequently entered into the model) on survival outcomes. We log (10)-transformed ferritin and sCD25 variables because they were skewed. Restricted cubic splines with three knots placed at the 10th, 50th and 90th percentiles (the number of knots was selected according to the Akaike information criterion) were generated to examine them after adjusting for confounding factors to examine whether nonlinear relationships existed between the ALBI score and the risk of mortality, and the tests for nonlinearity were calculated by Wald χ2 tests. The predictive powers of the ALBI score, serum ferritin, and their combination were evaluated using receiver operator characteristic (ROC) curve analysis.

All statistical analyses were performed with R software version 3.6.0 (R Foundation for Statistical Computing, Vienna, Austria) and STATA/MP statistical software (version 14.0; StataCorp, TX, USA), and two-sided *P* values < 0.05 were considered statistically significant.

## Results

### Baseline characteristics

**Figure 1:**
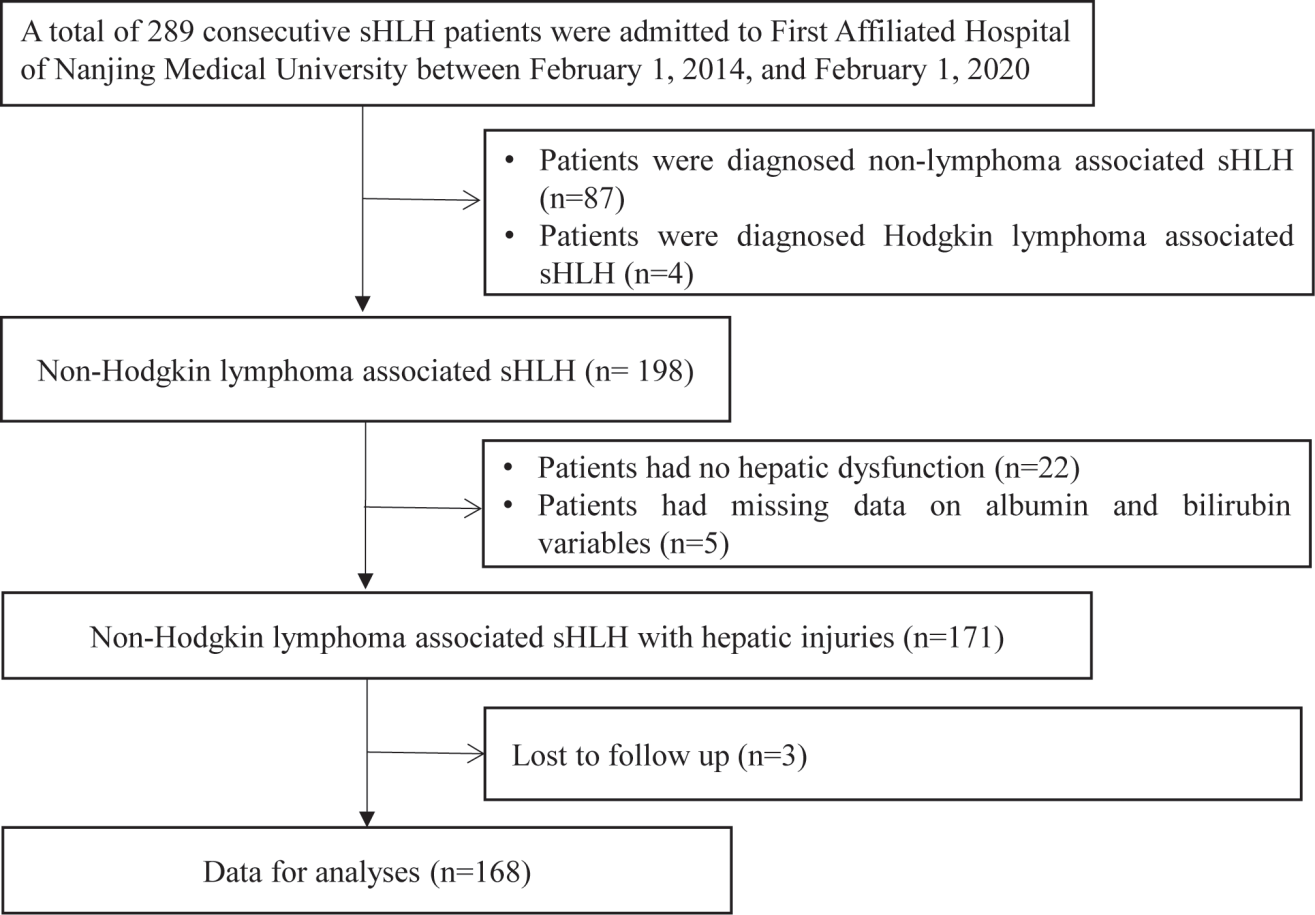
Flow diagram of the study population. sHLH, secondary hemophagocytic lymphohistiocytosis.

According to the protocol (**Figure 1**), 168 patients were ultimately included. Detailed demographic and clinical features of the NHL-sHLH patients with hepatic injuries are summarized in **Table 1**. The majority of patients were male (70.8%). The median age was 54 years (range 41–64 years) at the time of sHLH diagnosis. According to the sHLH etiologies (1), T cell or NK/T cell lymphoma (106, 63.1%) was the most frequent underlying disease. Stratification by ALBI grade identified 83 patients with ALBI grade 2 (49.4%), 83 patients with ALBI grade 3 (49.4%), and only 2 patients with ALBI grade 1 (1.2%). Owing to a small number of patients with ALBI grade 1, they were combined with the patients with ALBI grade 2.

**Table 1:** Baseline characteristics of NHL-sHLH patients with hepatic injuries.

Characteristics	Value (N=168)
Age (years)	54 (41-64)
Sex (M: F)	119: 49
ANC, ×10^9^/L	1.27 (0.62-2.00)
< 1.0	68 (40.5)
≥ 1.0	100 (59.5)
HB, g/L	81.50 (69.00-96.00)
< 90	106 (63.1)
≥ 90	62 (36.9)
PLT,×10^9^/L	39.00 (20.00-69.75)
< 100	149 (88.7)
≥ 100	19 (11.3)
ALB, g/L	27.22 ± 4.94
TBIL, umol/L	19.25 (13.10-39.70)
ALT, U/L	54.25 (32.80-111.85)
AST, U/L	86.85 (44.63-198.18)
LDH, U/L	740.00 (412.50-1404.25)
TG, mmol/L	2.46 (1.74-3.73)
< 3.0	102 (60.7)
≥ 3.0	66 (39.3)
FIB, g/L	1.44 (0.99-2.20)
≤ 1.5	90 (53.6)
> 1.5	78 (46.4)
Ferritin, ug/L	4290.50 (1506.00-13927.50)
sCD25, ng/L	40587.00 (22768.00-52839.00)
Splenomegaly, n (%)	157 (93.5)
Hepatomegaly, n (%)	63 (37.5)
Hemophagocytic, n (%)	145 (86.3)
HScore, points	237 (203-274)
EBV infection, n (%)	92 (54.8)
Type of lymphoma, n (%)	
B-sHLH	62 (36.9)
T/NKT-sHLH	106 (63.1)
Stage III/IV, n (%)	151(89.9)
B symptoms, n (%)	160 (95.2)
IPI score	3.3 ± 0.7
ALBI grade, n (%)	
1	2 (1.2)
2	83 (49.4)
3	83 (49.4)

ANC, absolute neutrophil count; HB, hemoglobin; PLT, platelet; ALB, albumin; TBIL, Total bilirubin; ALT, alanine transaminase; AST, aspartate transaminase; LDH, lactate dehydrogenase; TG, triglyceride; FIB, fibrinogen; sCD25, soluble interleukin-2 receptor; EBV, Epstein-Barr virus; B-sHLH, B cell lymphoma associated haemophagocytic lymphohistiocytosis; T/NKT-sHLH, T cell lymphoma or NK/T cell lymphoma associated haemophagocytic lymphohistiocytosis; ALBI, albumin- bilirubin; PALBI, platelet- albumin- bilirubin.

Data are means ± standard deviation or medians, with interquartile range in parentheses.

### Association between ALBI and study outcomes

**Figure 2:**
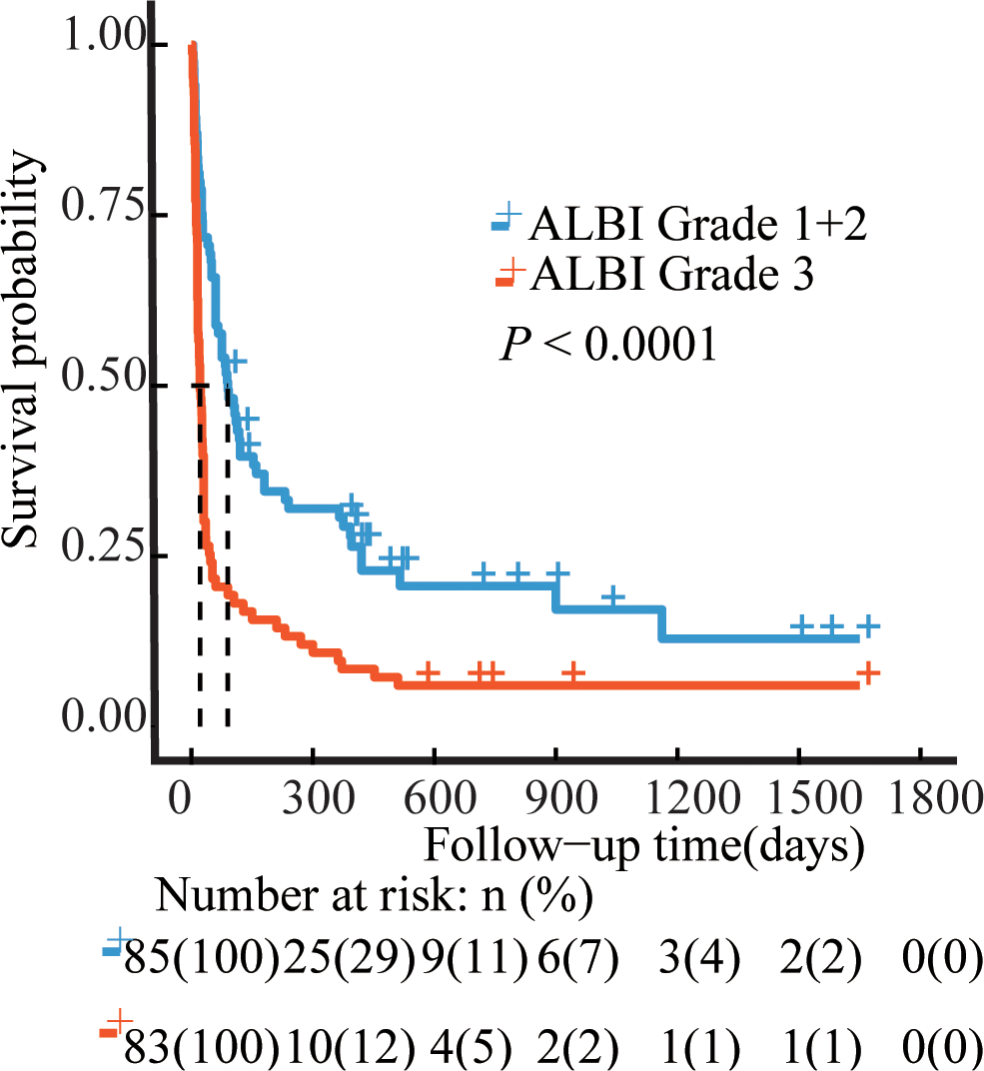
Survival curves of the NHL-sHLH patients according to ALBI grade. Blue line, ALBI grade 1 + 2 patients (N=85); Red line, ALBI grade 3 patients (N=83). P values were calculated by the log-rank test. ALBI, albumin-bilirubin.

**Figure 3:**
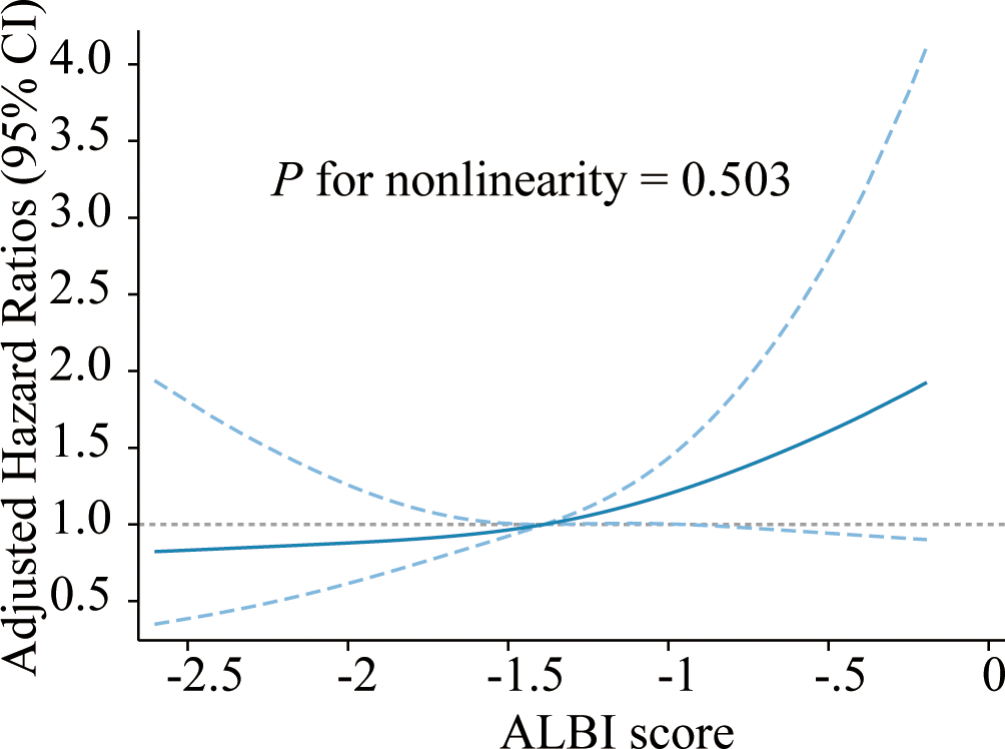
Cubic spline plot of the association between the ALBI score and risk of mortality among adult NHL-sHLH patients. The blue solid line and dashed line represent the estimated HRs and their corresponding 95% CIs, respectively. The gray dashed line is a reference line for HR=1. Analyses were adjusted for PLT, AST, FIB, TG, hepatomegaly, ferritin, etiologies, and treatment strategies. ALBI, albumin-bilirubin; NHL, non-Hodgkin lymphoma; HLH, hemophagocytic lymphohistiocytosis; PLT, platelet; AST, aspartate transaminase; FIB, fibrinogen; TG, triglyceride; HR, hazard ratio; CI, confidence interval.

The median OS was 36 days (95% CI 15–175 days), 144 patients died, and 24 survived through the entire follow-up period. Factors that were found to be significantly associated with 30-day mortality and overall survival are listed in **Tables 2**, **3** according to univariable analysis (*P* < 0.05). After adjusting for variables including laboratory examinations and traditional HLH risk factors, ALBI grade 3 was identified as an independent predictor of 30-day mortality (OR = 5.37, 95% CI: 2.41- 12.64, *P* < 0.001) and overall survival (HR = 1.52, 95% CI: 1.06-2.18, *P* = 0.023), and ferritin was also independently associated with an increased risk of 30-day mortality (OR = 4.13, 95% CI: 1.73-10.42, *P* = 0.002) and overall survival (HR = 1.68, 95% CI: 1.12-2.53, *P* = 0.012). The Kaplan-Meier survival analysis showed that the median OS was 90.0 days for patients with ALBI grade 1/2 and 21.0 days for ALBI grade 3 (*P* < 0.0001) (**Figure 2**). When the ALBI score was analyzed as a continuous variable and controlling for the same variables, the estimated risk associated with mortality increased incrementally at gradually increasing scores (*P* for nonlinearity = 0.503, **Figure 3**).

**Table 2:** Univariate and multivariate analysis of predictors for 30 days mortality.

Variables (Ref)	Univariable analysis		Multivariable analysis	
	HR (95% CI)	*P*	HR (95% CI)	*P*
Male (female)	0.63 (0.32-1.24)	0.185		
Age (≤60 years)	0.97 (0.52-1.82)	0.933		
ANC (≥1.0×10^9^/L)	1.32 (0.71-2.45)	0.377		
HB (≥90g/L)	0.58 (0.31-1.10)	0.094		
PLT (≥100×10^9^/L)	6.02 (1.68-21.53)	**0.006**		
FIB (>1.5g/L)	1.80 (0.97-3.32)	0.061		
TG (<3.0mmol/L)	1.79 (0.96-3.35)	0.069		
LDH (≤2.5×ULN)	1.35 (0.73-2.48)	0.338		
ALT (≤2.5×ULN)	1.32 (0.63-2.79)	0.467		
AST (≤2.5×ULN)	2.51 (1.35-4.69)	**0.004**		
Splenomegaly	1.19 (0.78-2.01)	0.078		
Hepatomegaly	2.39 (1.14-5.00)	**0.021**		
Hemophagocytosis	0.86 (0.36-2.06)	0.728		
Log_10_Ferritin ug/L	5.07 (2.55-10.10)	**< 0.001**	4.13 (1.73-10.42)	**0.002**
Log_10_sCD25 ng/L	2.46 (0.97-6.25)	0.059		
EBV (no infection)	1.11 (0.61-2.04)	0.734		
T/NKT-sHLH (B-sHLH)	2.65 (1.38-5.08)	**0.003**		
HLH04/Chemotherapy (GC±VP16±IVIg)	0.13 (0.06-0.31)	**< 0.001**	0.23 (0.08, 0.59)	**0.004**
ALB, g/L	0.07 (0.01-0.40)	**0.003**		
TBIL, umom/L	2.23 (1.50-3.32)	**< 0.001**		
ALBI				
grade 3 (grade 1+2)	5.90 (3.07-11.67)	**< 0.001**	5.37 (2.41-12.64)	**<0.001**

ANC, absolute neutrophil count; HB, hemoglobin; PLT, platelet; ALB, albumin; TBIL, Total bilirubin; ALT, alanine transaminase; AST, aspartate transaminase; LDH, lactate dehydrogenase; TG, triglyceride; FIB, fibrinogen; sCD25, soluble interleukin-2 receptor; EBV, Epstein-Barr virus; B-sHLH, B cell lymphoma associated haemophagocytic lymphohistiocytosis; T/NKT-sHLH, T cell lymphoma or NK/T cell lymphoma associated haemophagocytic lymphohistiocytosis; GC, glucocorticoid; IVIg, intravenous immunoglobulins; VP16, etoposide; ALBI, albumin- bilirubin.

OR, odds ratio; 95% CI, 95% confidence interval.

Bold statistical significance.

**Table 3:** Univariate and multivariate analysis of predictors for overall survival.

Variables (Ref)	Univariable analysis		Multivariable analysis	
	HR (95% CI)	*P*	HR (95% CI)	*P*
Male (female)	1.15 (0.80-1.66)	0.441		
Age (≤60 years)	1.01 (0.72-1.42)	0.956		
ANC (≥1.0×109/L)	1.20 (0.86-1.67)	0.289		
HB (≥90g/L)	1.25 (0.89-1.77)	0.200		
PLT (≥100×109/L)	2.25 (1.24-4.07)	**0.007**	1.94 (1.03-3.63)	**0.040**
FIB (>1.5g/L)	1.61 (1.15-2.25)	**0.005**		
TG (<3.0mmol/L)	1.46 (1.04-2.03)	**0.027**		
LDH (≤2.5×ULN)	1.26 (0.91-1.76)	0.164		
ALT (≤2.5×ULN)	1.08 (1.72-0.63)	0.697		
AST (≤2.5×ULN)	1.55 (1.11-2.16)	**0.009**		
Splenomegaly	1.47 (0.87-2.31)	0.069		
Hepatomegaly	1.46 (1.00-2.14)	**0.049**		
Hemophagocytosis	1.17 (0.72-1.90)	0.522		
Log10Ferritin ug/L	2.32 (1.63-3.32)	**< 0.001**	1.68 (1.12-2.53)	**0.012**
Log10sCD25 ng/L	1.69 (0.99-2.88)	0.051		
EBV (no infection)	1.36 (0.97-1.89)	0.074		
T/NKT-sHLH (B-sHLH)	1.86 (1.31-2.65)	**< 0.001**	1.53 (1.03-2.25)	**0.033**
HLH04/Chemotherapy (GC±VP16±IVIg)	0.31(0.21-0.45)	**< 0.001**	0.36 (0.24, 0.53)	**<0.001**
ALB g/L	0.29 (0.12-0.70)	**0.006**		
TBIL umom/L	1.58 (1.32-1.89)	**< 0.001**		
ALBI				
grade 3 (grade 1+2)	2.15 (1.54-2.99)	**< 0.001**	1.52 (1.06-2.18)	**0.023**

ANC, absolute neutrophil count; HB, hemoglobin; PLT, platelet; ALB, albumin; TBIL, Total bilirubin; ALT, alanine transaminase; AST, aspartate transaminase; LDH, lactate dehydrogenase; TG, triglyceride; FIB, fibrinogen; sCD25, soluble interleukin-2 receptor; EBV, Epstein-Barr virus; B-sHLH, B cell lymphoma associated haemophagocytic lymphohistiocytosis; T/NKT-sHLH, T cell lymphoma or NK/T cell lymphoma associated haemophagocytic lymphohistiocytosis; GC, glucocorticoid; IVIg, intravenous immunoglobulins; VP16, etoposide; ALBI, albumin- bilirubin.

OR, odds ratio; HR, hazards ratio; 95% CI, 95% confidence interval.

Bold statistical significance

### The ALBI score improves risk prediction according to time-dependent ROC analysis

**Figure 4:**
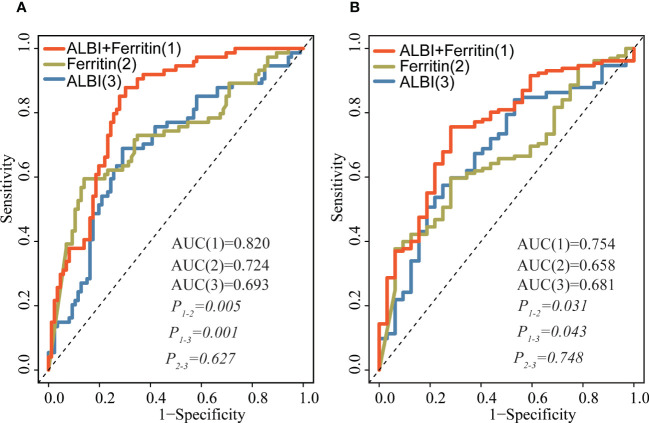
Time-dependent ROC analysis for the ALBI score, ferritin and their combination in predicting survival at 30 days **(A)** and after 1 year **(B)** of follow-up. Prediction performance was based on ROC. The red lines represent the ROC curve of ALBI and ferritin, and the blue and yellow lines represent the ROC curves of ALBI, ferritin, respectively. ROC, receiver operator curves; ALBI, albumin-bilirubin.

**Figure 4** shows the AUCs for the ALBI score and serum ferritin levels at 30 days and 1 year after the start of follow-up using time-dependent ROC analysis. Ferritin combined with the ALBI score showed the highest accuracy in predicting 30-day and 1-year mortality compared to either the ferritin (AUC_30 days_: 0.820 vs 0.724, *P* = 0.005; AUC_1 year_: 0.754 vs 0.658, *P* = 0.031) or ALBI score (AUC _30 days_: 0.820 vs 0.693, *P* = 0.001; AUC_1 year_: 0.754 vs 0.681, *P* = 0.043) alone.

In addition, **Supplementary Figure 1** shows the liver PET-CT images of two NHL-sHLH patients with different ALBI scores before treatment. Patient 1 had ALBI score grade 1, and patient 2 had ALBI score grade 3. In our case, the liver was enlarged in patient 2, and the SUVmax in the liver was significantly higher than patients 1. Favorable long-term survival was reported in the patient 1 after treatment. However, patient 2 died 28 days after disease onset.

## Discussion

In this retrospective study, the ALBI score was associated with an increased risk of mortality among NHL-sHLH patients with hepatic injuries. The combination of pretreatment ALBI score and serum ferritin, a benchmark prognostic marker in sHLH patients, improved prognostic prediction discrimination. Our data revealed that ALBI was a better prognostic tool in predicting short-term (30 days) or long-term (≥1 year) survival, which persisted after extensive adjustment of confounding variables, including demographic, clinical, laboratory examinations and traditional sHLH risk factors. Moreover, we demonstrated that the ALBI score has a direct positive association with mortality in the total cohort by restricted cubic spline (RCS) modeling. In addition, the combination of the ALBI score and ferritin provided incremental prognostic value for each measure separately for both 30-day and 1-year survival. These results suggest that the ALBI score is a better predictive marker for survival in patients with hepatic injuries.

Lymphoma is a common trigger identified in adult sHLH, especially non-Hodgkin lymphoma-associated sHLH (NHL-sHLH), which has a high mortality rate, with an estimated 30-day survival of approximately 56-70% and 2-year survival of 8-34.3% (21–23). These patients frequently present a combination of persistent fever, elevated aminotransferase (AST, ALT), decreased serum albumin, jaundice, and hepatomegaly or splenomegaly. Several studies have evaluated whether sHLH patients with hepatic involvement have a poorer prognosis (8, 14); therefore, early prediction of survival outcome contributes greatly to patient management and aids in making appropriate risk assessments as well as treatment decisions. Clinically, the ALBI score has been widely used for assessing liver function and predicting survival outcomes in various diseases with liver dysfunction. Takeshi et al. prospectively evaluated the ALBI grade as a predictor of survival in a multicenter cohort of 1,190 patients with acute heart failure (AHF). After adjusting for pre-existing prognostic factors, the ALBI score was associated with 1-year mortality (HR= 2.11, 95% CI: 1.60–2.79, *P*<0.001) (12). Zhang also showed similar findings; the ALBI grade appears to be a promising prognostic biomarker associated with overall survival (HR=1.60, 95% CI: 1.12–2.29, *P*=0.02) in critically ill patients with acute pancreatitis (13). Moreover, several studies have described that serum albumin and total bilirubin are significantly associated with mortality in HLH patients (8, 24). Our study, by Multiple analysis and RCS modeling, clearly demonstrated that ALBI was strongly associated with survival outcomes (both 30-day mortality and overall survival (≥ 1 year)). In addition, our investigation in this study demonstrated that the combination analysis of ALBI score and ferritin had a significant improvement over either ALBI score (AUC _30 days_: 0.820 vs 0.693, *P* = 0.001; AUC_1 year_: 0.754 vs 0.681, *P* = 0.043) or ferritin (AUC_30 days_: 0.820 vs 0.724, *P* = 0.005; AUC_1 year_: 0.754 vs 0.658, *P* = 0.031) alone.

The increased risk of mortality associated with a higher ALBI score might be ascribed to uncontrolled proliferation of cytotoxic CD8+ T lymphocytes (CTLs) and macrophages, creating an uncontrolled loop of inflammation that is responsible for hepatic injuries. ALBI, consisting of serum albumin and total bilirubin, could reflect the systemic inflammatory response. On the one hand, serum albumin plays an important role in modulating systemic inflammatory reactions and organic oxidation resistance (25); on the other hand, serum total bilirubin is always elevated with hepatobiliary dysfunction in response to various cytokines in circulating blood, such as interferon-g (IFN-γ), interleukin-1beta (IL-1*β*), and interleukin-6 (IL-6) (8). All of the above mechanisms, alone or together, may be attributed to decreased serum albumin and elevated total bilirubin and indirectly reflect the degree of hepatic injury.

Our findings provide references that routine clinical laboratory assays such as serum albumin and total bilirubin can be used to identify patients at higher risk of death while applying an advanced evaluation for NHL-sHLH patients. This is the first study that addressed the ALBI score, a promising hepatic injury tool, and the risk of mortality (both short-term and long-term survival). The ALBI score improved risk predictions of mortality in both the short and long term and could provide clinical guidance for timely multidisciplinary discussions of when to initiate HLH-directed immune suppressants and NHL-directed intensive chemotherapy. However, several limitations of our study were noted. First, the enrolled study population was small, and the study was retrospective in nature. Second, although we had fully adjusted a broad set of covariates, we could not rule out the role of unmeasured or unrecognized confounders. Third, NGS on lymphoma or all sHLH patients were not routinely measured at admission due to economic costs. Finally, the performance of the ALBI was not confirmed in the internal and external validation cohorts. Thus, prospective and multicenter research is needed in the future.

## Conclusion

Our study demonstrated that the ALBI score was associated with 30-day mortality and long-term (≥ 1 year) survival among NHL-sHLH patients with hepatic injuries. Early detection based on a higher ALBI score/risk grade could be helpful in ensuring prompt interventions to reduce mortality.

## Data availability statement

The original contributions presented in the study are included in the article/**Supplementary Material**. Further inquiries can be directed to the corresponding authors.

## Ethics statement

Our study was approved by the ethics committee of the First Affiliated Hospital of Nanjing Medical University and registered on the Chinese Clinical Trial Registry (ChiCTR2000032421). Written informed consent for participation was not required for this study in accordance with the national legislation and the institutional requirements.

## Author contributions

GY, WC, JX and HQ designed the experiment. GY, YS and WC performed the experiments. GY, WC, JX, LD and HQ organized the clinical materials. JX, LD and WC performed the data analysis and wrote the paper. All authors contributed to the final approval of the manuscript.
