# Supplementary material for: Prognostic value of the albumin-bilirubin score in patients with non-Hodgkin lymphoma-associated hemophagocytic lymphohistiocytosis
Source: Front Immunol. 2023 May 17;14:1162320. doi: 10.3389/fimmu.2023.1162320 (PMC10229876; doi:10.3389/fimmu.2023.1162320)
Supplement: Supplementary file 1 [file DataSheet_1.zip › Supplementary Table 1.DOCX]

The patient characteristics differed between the different ALBI groups was showed as follows (Supplementary Table 1) :

Supplementary Table 1. Baseline characteristics of NHL-sHLH patients according to ALBI score

| Variable | ALL | ALBI SCORE | | *P* VALUE |
| --- | --- | --- | --- | --- |
|  |  | grade 1+2 | grade 3 |  |
| No. of patients (n) | 168 | 85 | 83 |  |
| Male,n(%) | 119 (70.8) | 54 (63.5) | 65 (78.3) | 0.036^*^ |
| Age, years | 61(48-68) | 51 (35-63) | 58 (47-66) | 0.027^*^ |
| ANC, 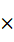10^9^/L | 1.27 (0.62-2.00) | 1.16 (0.52-1.86) | 1.32 (0.75-2.31) | 0.093 |
| HB, g/L | 81.00 (69.00-96.00) | 85.00 (72 .00- 107.00) | 79.00 (65.50-92.00) | 0.034^*^ |
| PLT,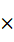10^9^/L | 39.00 (20.00-69.75) | 46.00(27.00- 84.00) | 30.00 (12.50- 48.00) | <0.001^***^ |
| FIB, g/L | 1.44 (0.99-2.20) | 1.54(1.06- 2.31) | 1.35 (0.92-2.10) | 0.196 |
| TG, mmol/L | 2.46 (1.74-3.73) | 2.44(1.63- 3.43) | 2.55 (1.92-3.85) | 0.116 |
| ALB, g/L | 27.22 ± 4.94 | 30.1(27.8- 33.4) | 23.3 (21.3- 26.3) | <0.001^***^ |
| LDH, U/L | 740.00 (412.50-1404.25) | 683.00(461.00-1252.00) | 775.00 (403.50- 1495.00) | 0.439 |
| ALT, U/L | 54.25 (32.80-111.85) | 53.6 (32.8-97.7) | 59.4 (43.6-117.4) | 0.531 |
| AST, U/L | 86.85 (44.63-198.18) | 69.6 (39.3-97.7) | 112.8 (67.0-218.1) | 0.002^**^ |
| TBIL, umol/L | 19.25 (13.10-39.70) | 15.00 (10.5-19.6) | 35.9 (19.0-79.1) | <0.001^***^ |
| ferritin, ug/L | 4290.50  (1506.00-13927.50) | 3639.50  (1604.00-13131.10) | 5391.00  (1503.00-16200) | 0.470 |
| sCD25, ng/L | 40587.00  (22768.00-52839.00) | 36590.00  (22660.00-48389.00) | 43172  (23701.50-57508.00) | 0.125 |
| Splenomegaly,n(%) | 157 (93.5) | 77 (90.6) | 80 (70.8) | 0.130 |
| Hepatomegaly, n (%) | 63 (37.5) | 23 (27.1) | 40 (48.2) | 0.045^*^ |
| Hemophagocytic,n (%) | 145 (86.3) | 77(79.7) | 68 (81.9) | 0.104 |
| EBV infection, n (%) | 92 (54.8) | 45 (90.6) | 47 (56.6) | 0.632 |

Abbreviations: ANC, absolute neutrophil count; HB, hemoglobin; PLT, platelet; FIB, fibrinogen; TG, triglyceride; ALB, albumin; LDH, lactate dehydrogenase; ALT, alanine transaminase; AST, aspartate transaminase; TBIL, total bilirubin; sCD25, soluble interleukin -2 receptor; EBV, Epstein-Barr virus.

**P* < 0.05, ***P* < 0.01, ****P* < 0.001 when compared with the normal group.
