# Supplementary material for: Prognostic value of the albumin-bilirubin score in patients with non-Hodgkin lymphoma-associated hemophagocytic lymphohistiocytosis
Source: Front Immunol. 2023 May 17;14:1162320. doi: 10.3389/fimmu.2023.1162320 (PMC10229876; doi:10.3389/fimmu.2023.1162320)
Supplement: Supplementary file 1 [file DataSheet_1.zip › Supplementary Table 2.DOCX]

The patient characteristics differed between the NHL-sHLH and NHL with LFT/Albumin abnormalities groups was showed as follows (Supplementary Table 2):

Supplementary Table 2. Baseline characteristics of patients in NHL-sHLH group and NHL with LFT/Albumin abnormalities group

| Variable | NHL with HLH | NHL with LFT/Albumin abnormalities | *P* VALUE |
| --- | --- | --- | --- |
| No. of patients (n) | 168 | 206 |  |
| Male,n(%) | 119 (70.8) | 86 (41.7) | 0.648 |
| Age (years), median, range | 61(48-68) | 67 (57-74) | 0.512 |
| ANC<1.5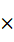10^9^/L, n (%) | 93 (55.4) | 6 (2.9) | <0.001^***^ |
| HB<90 g/L, n (%) | 106 (63.1) | 13 (6.3) | <0.001^***^ |
| PLT<100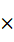10^9^/L, n (%) | 149 (88.7) | 5 (2.4) | <0.001^***^ |
| FIB<1.5 g/L, n (%) | 90 (53.6) | 3 (1.5) | <0.001^***^ |
| TG>3 mmol/L, n (%) | 66 (39.3) | 25 (12.1) | 0.131 |
| LDH>240 U/L, n (%) | 161 (95.8) | 76 (36.9) | 0.008 |
| ALB, g/L | 27.22 ± 4.94 | 30.12 ± 5.11 | <0.001^***^ |
| ALT, U/L | 54.25 (32.80-111.85) | 30.25 (12.28-49.65) | 0.015 |
| AST, U/L | 86.85 (44.63-198.18) | 41.10 (35.75-62.05) | 0.012 |
| TBIL, umol/L | 19.25 (13.10-39.70) | 17.25 (12.90-37.10) | 0.023 |
| ferritin, ug/L | 4290.50  (1506.00-13927.50) | 461.6  (137.84–719.10) | <0.001^***^ |
| sCD25, ng/L | 40587.00  (22768.00-52839.00) | 8953.00  (4345.00-91228.00) | <0.001^***^ |
| Splenomegaly,n(%) | 157 (93.5) | 32 (15.5) | <0.001^***^ |
| Hepatomegaly, n (%) | 63 (37.5) | 30 (14.6) | <0.001^***^ |
| Hemophagocytic,n (%) | 145 (86.3) | 0 | <0.001^***^ |
| EBV infection, n (%) | 92 (54.8) | 70 (33.9) | 0.061 |

Abbreviations: ANC, absolute neutrophil count; HB, hemoglobin; PLT, platelet; FIB, fibrinogen; TG, triglyceride; LDH, lactate dehydrogenase; ALB, albumin; ALT, alanine transaminase; AST, aspartate transaminase; TBIL, total bilirubin; sCD25, soluble interleukin -2 receptor; EBV, Epstein-Barr virus.

**P* < 0.05, ***P* < 0.01, ****P* < 0.001 when compared with the normal group.
