# Supplementary figures and images for: Prognostic value of the albumin-bilirubin score in patients with non-Hodgkin lymphoma-associated hemophagocytic lymphohistiocytosis
Source: Front Immunol. 2023 May 17;14:1162320. doi: 10.3389/fimmu.2023.1162320 (PMC10229876; doi:10.3389/fimmu.2023.1162320)

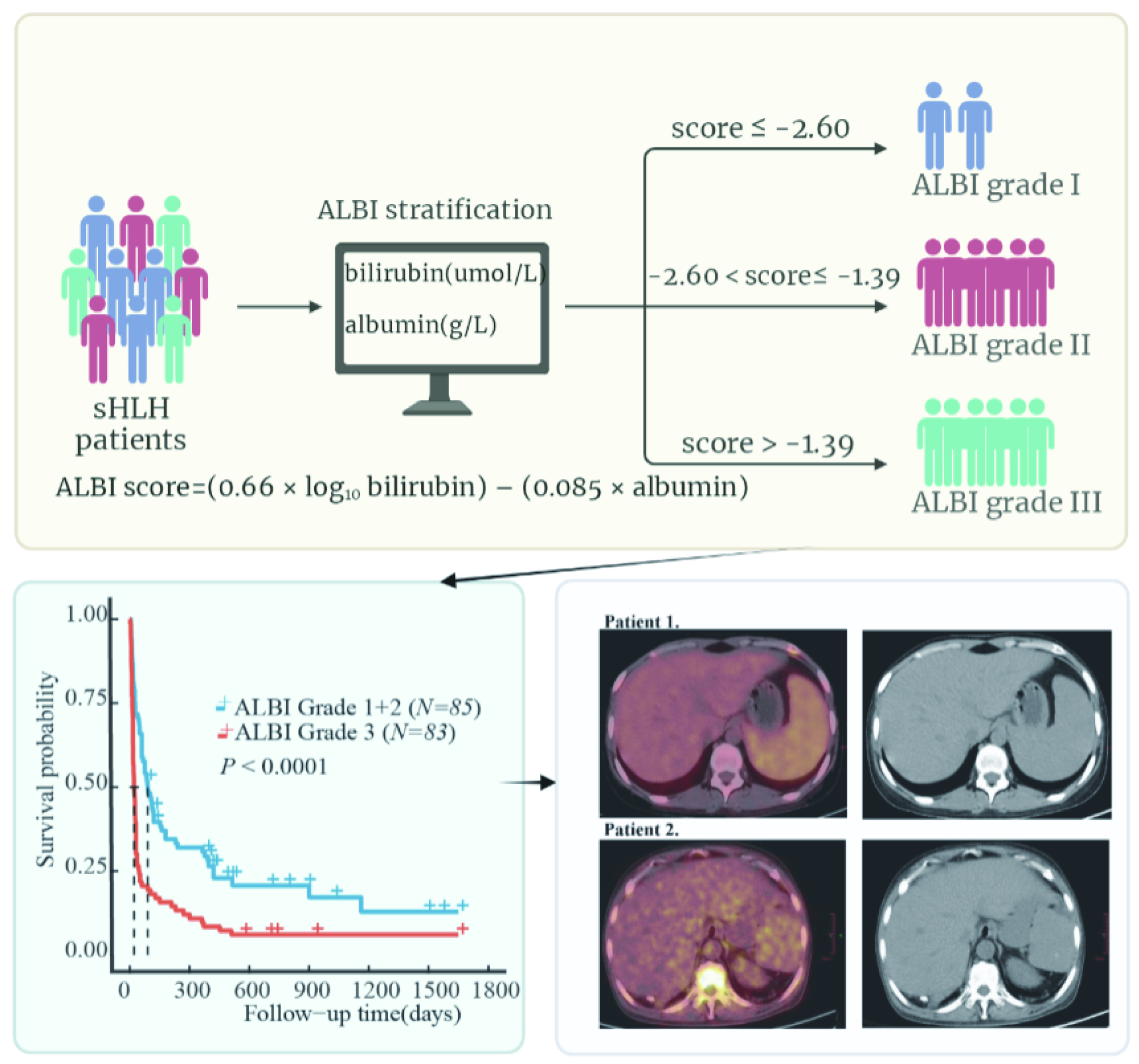

Supplement: Supplementary file 2 [file Image_1.tif]
